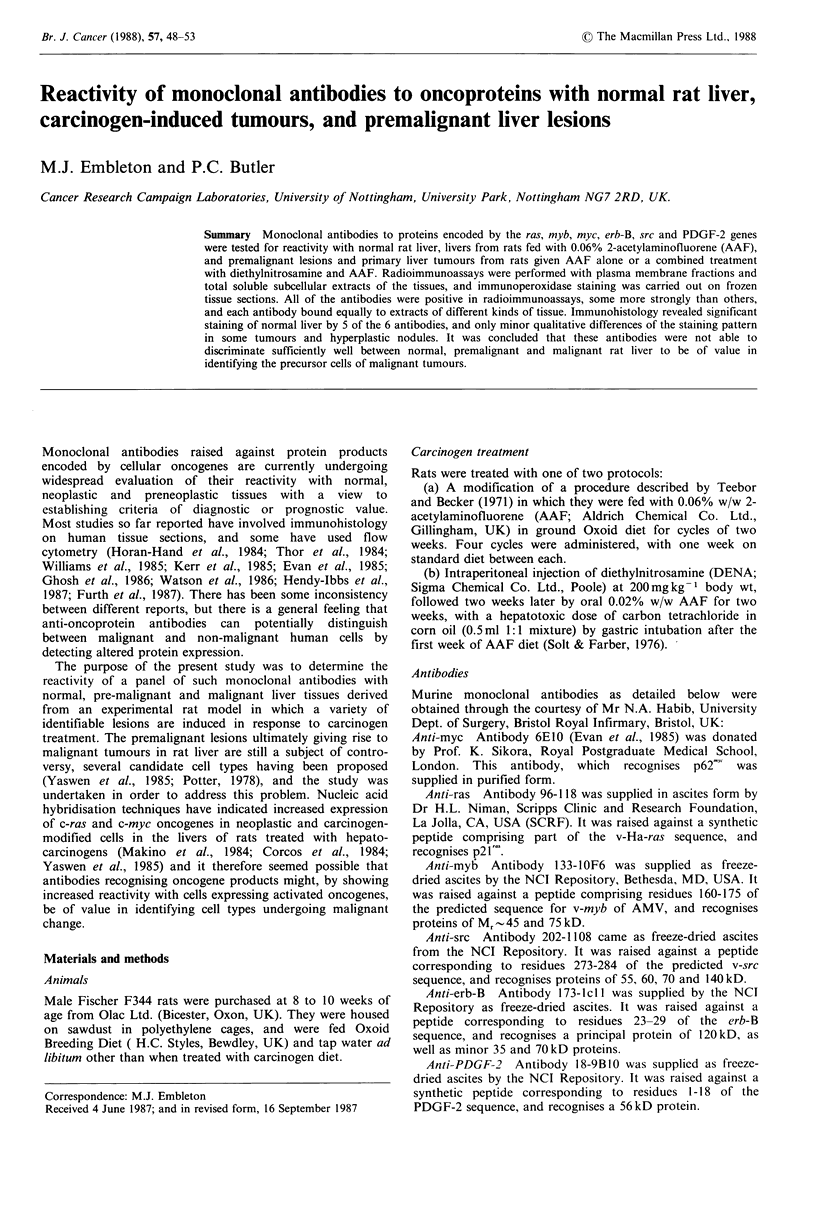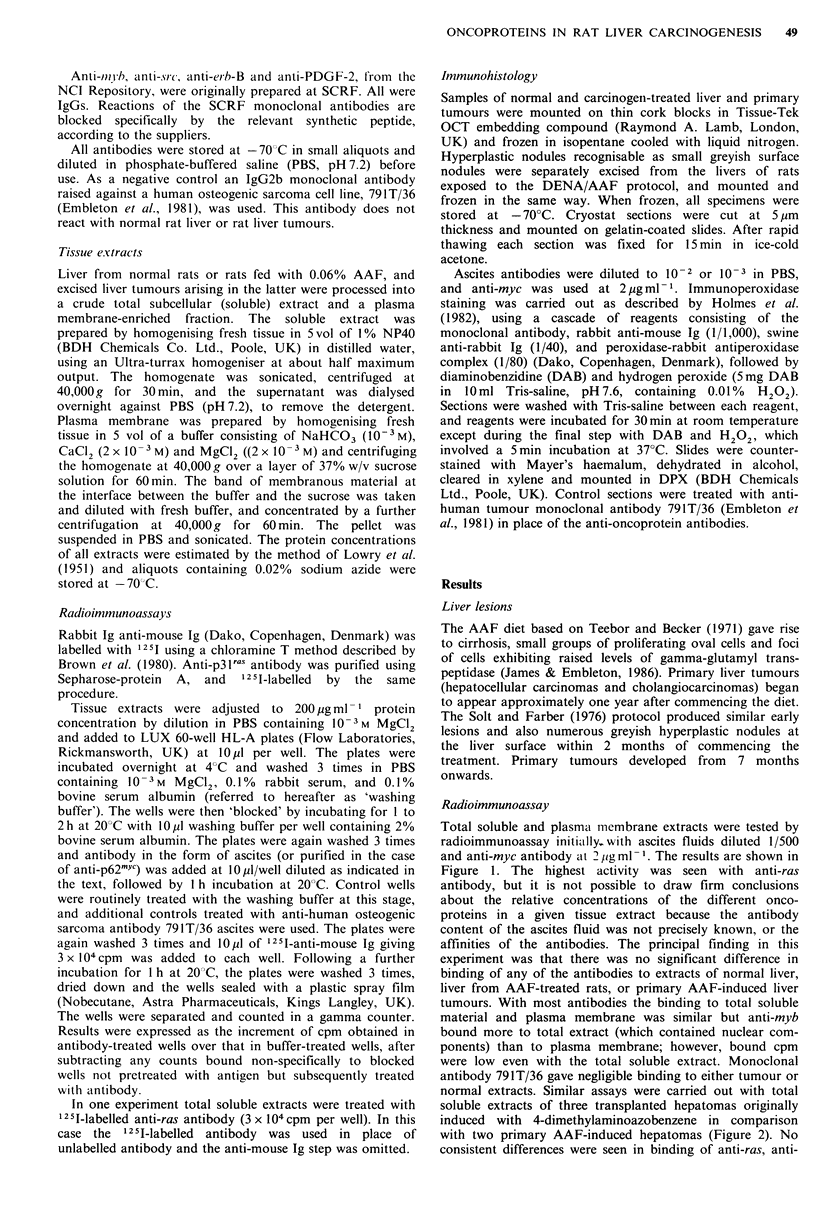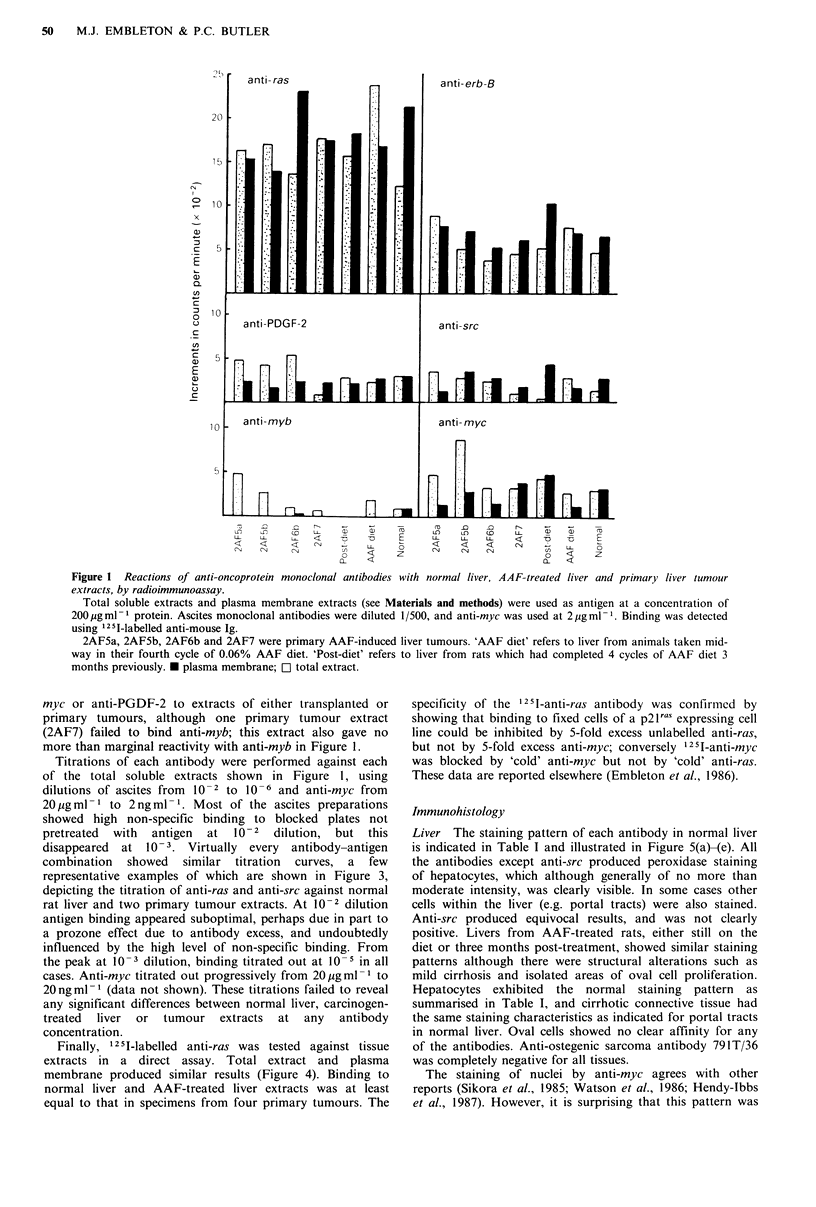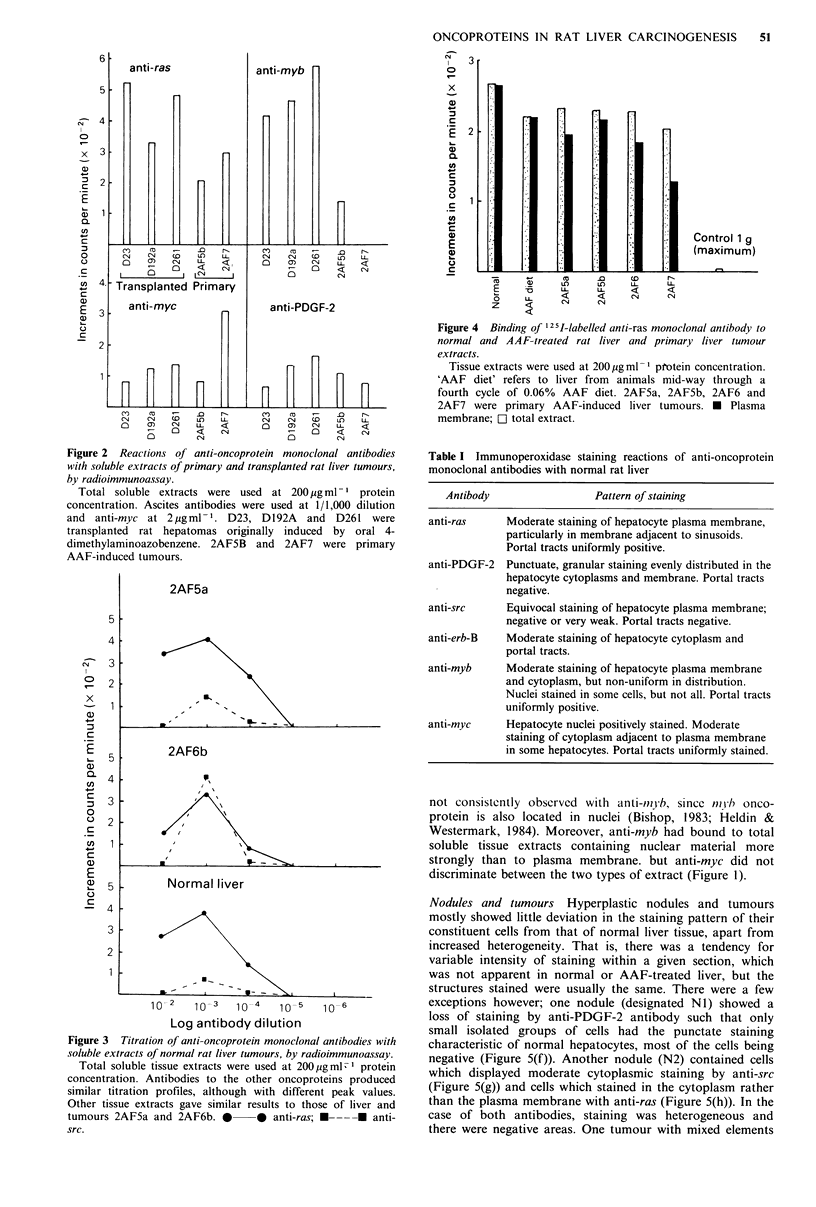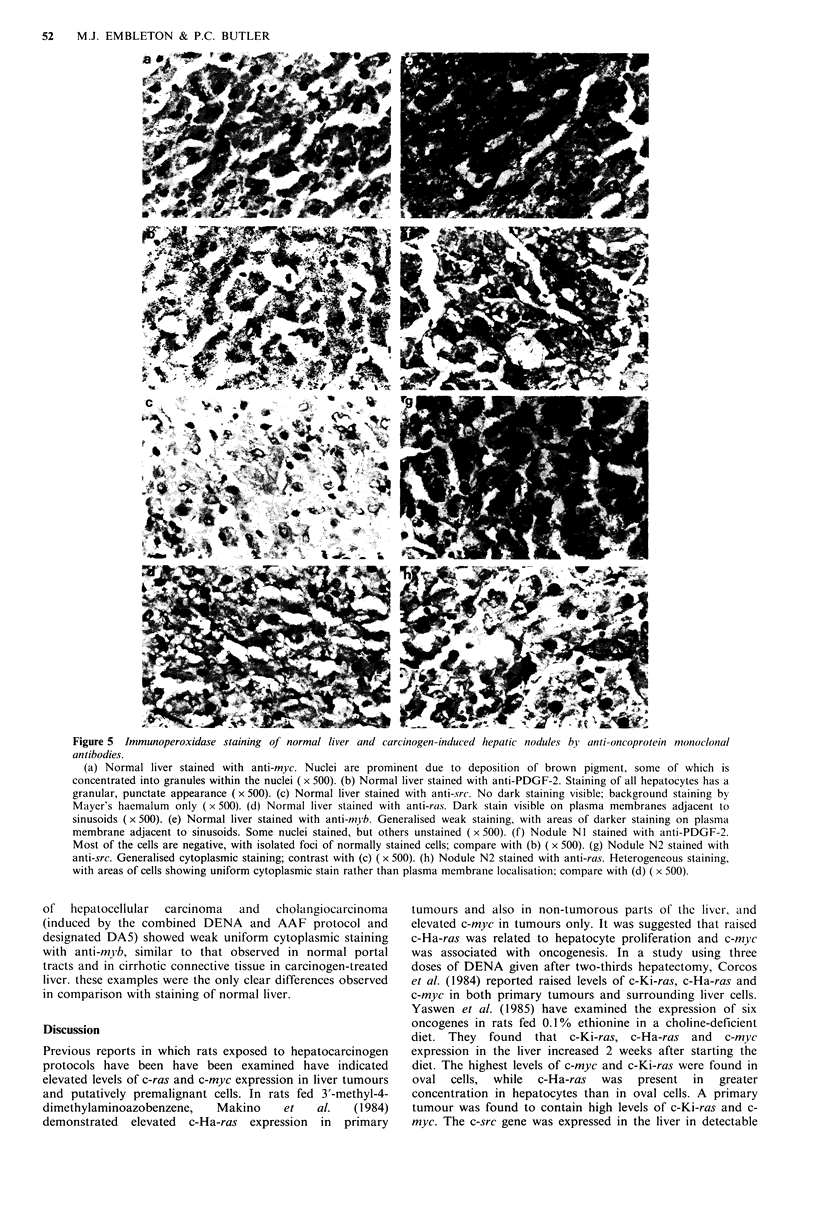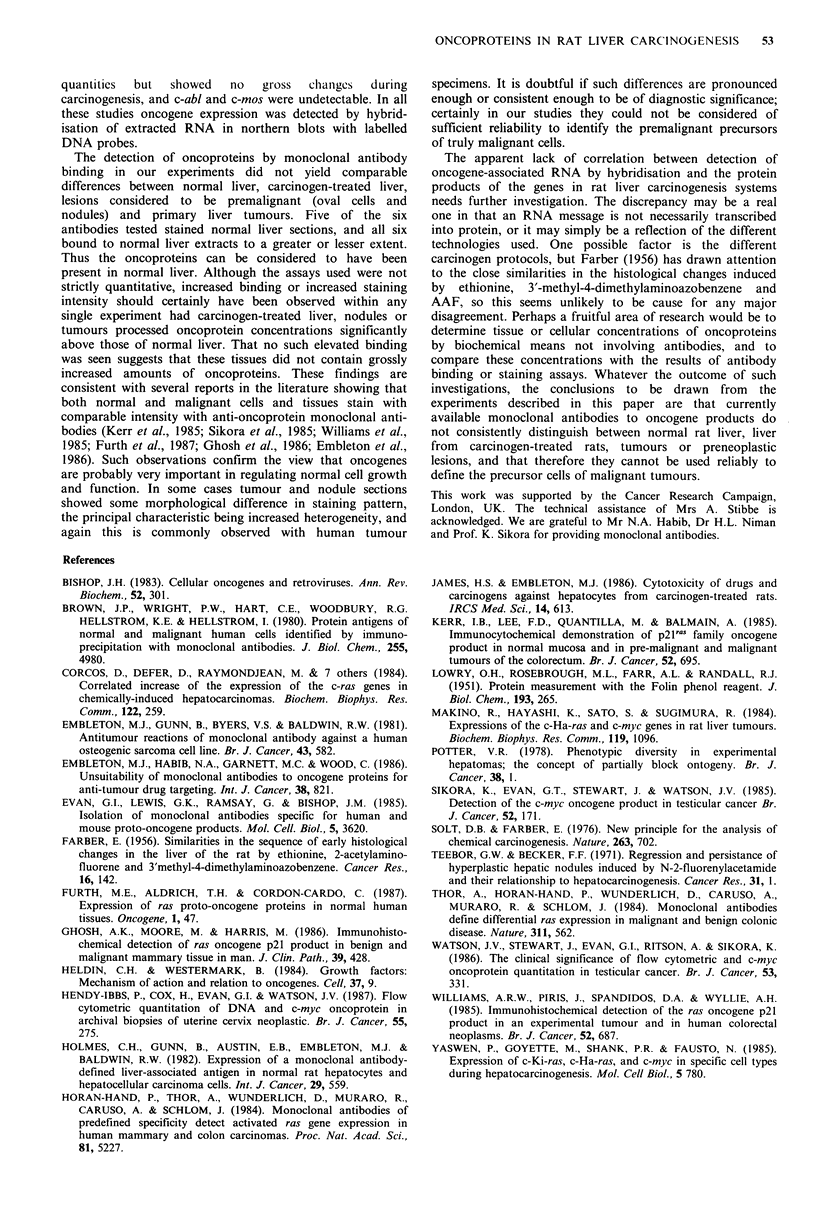# Reactivity of monoclonal antibodies to oncoproteins with normal rat liver, carcinogen-induced tumours, and premalignant liver lesions.

**DOI:** 10.1038/bjc.1988.7

**Published:** 1988-01

**Authors:** M. J. Embleton, P. C. Butler

**Affiliations:** Cancer Research Campaign Laboratories, University of Nottingham, UK.

## Abstract

**Images:**


*Br. J. Cancer* (1988), **57**, 48–53 © The Macmillan Press Ltd., 1988

### Reactivity of monoclonal antibodies to oncoproteins with normal rat liver, carcinogen-induced tumours, and premalignant liver lesions

M.J. Embleton and P.C. Butler

*Cancer Research Campaign Laboratories, University of Nottingham, University Park, Nottingham NG7 2RD, UK.*

**Summary** Monoclonal antibodies to proteins encoded by the *ras*, *myb*, *myc*, *erb*-B, *src* and PDGF-2 genes were tested for reactivity with normal rat liver, livers from rats fed with 0.06% 2-acetylaminofluorene (AAF), and premalignant lesions and primary liver tumours from rats given AAF alone or a combined treatment with diethylnitrosamine and AAF. Radioimmunoassays were performed with plasma membrane fractions and total soluble subcellular extracts of the tissues, and immunoperoxidase staining was carried out on frozen tissue sections. All of the antibodies were positive in radioimmunoassays, some more strongly than others, and each antibody bound equally to extracts of different kinds of tissue. Immunohistology revealed significant staining of normal liver by 5 of the 6 antibodies, and only minor qualitative differences of the staining pattern in some tumours and hyperplastic nodules. It was concluded that these antibodies were not able to discriminate sufficiently well between normal, premalignant and malignant rat liver to be of value in identifying the precursor cells of malignant tumours.

Monoclonal antibodies raised against protein products encoded by cellular oncogenes are currently undergoing widespread evaluation of their reactivity with normal, neoplastic and preneoplastic tissues with a view to establishing criteria of diagnostic or prognostic value. Most studies so far reported have involved immunohistology on human tissue sections, and some have used flow cytometry (Horan-Hand *et al.*, 1984; Thor *et al.*, 1984; Williams *et al.*, 1985; Kerr *et al.*, 1985; Evan *et al.*, 1985; Ghosh *et al.*, 1986; Watson *et al.*, 1986; Hendy-Ibbs *et al.*, 1987; Furth *et al.*, 1987). There has been some inconsistency between different reports, but there is a general feeling that anti-oncoprotein antibodies can potentially distinguish between malignant and non-malignant human cells by detecting altered protein expression.

The purpose of the present study was to determine the reactivity of a panel of such monoclonal antibodies with normal, pre-malignant and malignant liver tissues derived from an experimental rat model in which a variety of identifiable lesions are induced in response to carcinogen treatment. The premalignant lesions ultimately giving rise to malignant tumours in rat liver are still a subject of controversy, several candidate cell types having been proposed (Yaswen *et al.*, 1985; Potter, 1978), and the study was undertaken in order to address this problem. Nucleic acid hybridisation techniques have indicated increased expression of c-*ras* and c-*myc* oncogenes in neoplastic and carcinogen-modified cells in the livers of rats treated with hepatocarcinogens (Makino *et al.*, 1984; Corcos *et al.*, 1984; Yaswen *et al.*, 1985) and it therefore seemed possible that antibodies recognising oncogene products might, by showing increased reactivity with cells expressing activated oncogenes, be of value in identifying cell types undergoing malignant change.

### Materials and methods

### *Animals*

Male Fischer F344 rats were purchased at 8 to 10 weeks of age from Olac Ltd. (Bicester, Oxon, UK). They were housed on sawdust in polyethylene cages, and were fed Oxoid Breeding Diet ( H.C. Styles, Bewdley, UK) and tap water *ad libitum* other than when treated with carcinogen diet.

Correspondence: M.J. Embleton
Received 4 June 1987; and in revised form, 16 September 1987

### *Carcinogen treatment*

Rats were treated with one of two protocols:

(a) A modification of a procedure described by Teebor and Becker (1971) in which they were fed with 0.06% w/w 2-acetylaminofluorene (AAF; Aldrich Chemical Co. Ltd., Gillingham, UK) in ground Oxoid diet for cycles of two weeks. Four cycles were administered, with one week on standard diet between each.

(b) Intraperitoneal injection of diethylnitrosamine (DENA; Sigma Chemical Co. Ltd., Poole) at $200\,mg\,kg^{-1}$ body wt, followed two weeks later by oral 0.02% w/w AAF for two weeks, with a hepatotoxic dose of carbon tetrachloride in corn oil (0.5 ml 1:1 mixture) by gastric intubation after the first week of AAF diet (Solt & Farber, 1976).

### *Antibodies*

Murine monoclonal antibodies as detailed below were obtained through the courtesy of Mr N.A. Habib, University Dept. of Surgery, Bristol Royal Infirmary, Bristol, UK:

*Anti*-myc Antibody 6E10 (Evan *et al.*, 1985) was donated by Prof. K. Sikora, Royal Postgraduate Medical School, London. This antibody, which recognises $p62^{myc}$ was supplied in purified form.

*Anti*-ras Antibody 96-118 was supplied in ascites form by Dr H.L. Niman, Scripps Clinic and Research Foundation, La Jolla, CA, USA (SCRF). It was raised against a synthetic peptide comprising part of the v-Ha-*ras* sequence, and recognises $p21^{ras}$.

*Anti*-myb Antibody 133-10F6 was supplied as freeze-dried ascites by the NCI Repository, Bethesda, MD, USA. It was raised against a peptide comprising residues 160-175 of the predicted sequence for v-*myb* of AMV, and recognises proteins of $M_r \sim 45$ and 75 kD.

*Anti*-src Antibody 202-1108 came as freeze-dried ascites from the NCI Repository. It was raised against a peptide corresponding to residues 273-284 of the predicted v-*src* sequence, and recognises proteins of 55, 60, 70 and 140 kD.

*Anti*-erb-B Antibody 173-1c11 was supplied by the NCI Repository as freeze-dried ascites. It was raised against a peptide corresponding to residues 23–29 of the *erb*-B sequence, and recognises a principal protein of 120 kD, as well as minor 35 and 70 kD proteins.

*Anti-PDGF-2* Antibody 18-9B10 was supplied as freeze-dried ascites by the NCI Repository. It was raised against a synthetic peptide corresponding to residues 1-18 of the PDGF-2 sequence, and recognises a 56 kD protein.

Anti-*myb*, anti-*src*, anti-*erb*-B and anti-PDGF-2, from the NCI Repository, were originally prepared at SCRF. All were IgGs. Reactions of the SCRF monoclonal antibodies are blocked specifically by the relevant synthetic peptide, according to the suppliers.

All antibodies were stored at −70°C in small aliquots and diluted in phosphate-buffered saline (PBS, pH 7.2) before use. As a negative control an IgG2b monoclonal antibody raised against a human osteogenic sarcoma cell line, 791T/36 (Embleton *et al.*, 1981), was used. This antibody does not react with normal rat liver or rat liver tumours.

*Tissue extracts*

Liver from normal rats or rats fed with 0.06% AAF, and excised liver tumours arising in the latter were processed into a crude total subcellular (soluble) extract and a plasma membrane-enriched fraction. The soluble extract was prepared by homogenising fresh tissue in 5 vol of 1% NP40 (BDH Chemicals Co. Ltd., Poole, UK) in distilled water, using an Ultra-turrax homogeniser at about half maximum output. The homogenate was sonicated, centrifuged at 40,000 *g* for 30 min, and the supernatant was dialysed overnight against PBS (pH 7.2), to remove the detergent. Plasma membrane was prepared by homogenising fresh tissue in 5 vol of a buffer consisting of $NaHCO_3$ ($10^{-3}$ M), $CaCl_2$ ($2 \times 10^{-3}$ M) and $MgCl_2$ (($2 \times 10^{-3}$ M) and centrifuging the homogenate at 40,000 *g* over a layer of 37% w/v sucrose solution for 60 min. The band of membranous material at the interface between the buffer and the sucrose was taken and diluted with fresh buffer, and concentrated by a further centrifugation at 40,000 *g* for 60 min. The pellet was suspended in PBS and sonicated. The protein concentrations of all extracts were estimated by the method of Lowry *et al.* (1951) and aliquots containing 0.02% sodium azide were stored at −70°C.

*Radioimmunoassays*

Rabbit Ig anti-mouse Ig (Dako, Copenhagen, Denmark) was labelled with $^{125}I$ using a chloramine T method described by Brown *et al.* (1980). Anti-p31$^{ras}$ antibody was purified using Sepharose-protein A, and $^{125}I$-labelled by the same procedure.

Tissue extracts were adjusted to 200 $\mu g\,ml^{-1}$ protein concentration by dilution in PBS containing $10^{-3}$ M $MgCl_2$ and added to LUX 60-well HL-A plates (Flow Laboratories, Rickmansworth, UK) at 10 $\mu l$ per well. The plates were incubated overnight at 4°C and washed 3 times in PBS containing $10^{-3}$ M $MgCl_2$, 0.1% rabbit serum, and 0.1% bovine serum albumin (referred to hereafter as 'washing buffer'). The wells were then 'blocked' by incubating for 1 to 2 h at 20°C with 10 $\mu l$ washing buffer per well containing 2% bovine serum albumin. The plates were again washed 3 times and antibody in the form of ascites (or purified in the case of anti-p62$^{myc}$) was added at 10 $\mu l$/well diluted as indicated in the text, followed by 1 h incubation at 20°C. Control wells were routinely treated with the washing buffer at this stage, and additional controls treated with anti-human osteogenic sarcoma antibody 791T/36 ascites were used. The plates were again washed 3 times and 10 $\mu l$ of $^{125}I$-anti-mouse Ig giving $3 \times 10^4$ cpm was added to each well. Following a further incubation for 1 h at 20°C, the plates were washed 3 times, dried down and the wells sealed with a plastic spray film (Nobecutane, Astra Pharmaceuticals, Kings Langley, UK). The wells were separated and counted in a gamma counter. Results were expressed as the increment of cpm obtained in antibody-treated wells over that in buffer-treated wells, after subtracting any counts bound non-specifically to blocked wells not pretreated with antigen but subsequently treated with antibody.

In one experiment total soluble extracts were treated with $^{125}I$-labelled anti-*ras* antibody ($3 \times 10^4$ cpm per well). In this case the $^{125}I$-labelled antibody was used in place of unlabelled antibody and the anti-mouse Ig step was omitted.

*Immunohistology*

Samples of normal and carcinogen-treated liver and primary tumours were mounted on thin cork blocks in Tissue-Tek OCT embedding compound (Raymond A. Lamb, London, UK) and frozen in isopentane cooled with liquid nitrogen. Hyperplastic nodules recognisable as small greyish surface nodules were separately excised from the livers of rats exposed to the DENA/AAF protocol, and mounted and frozen in the same way. When frozen, all specimens were stored at −70°C. Cryostat sections were cut at 5 $\mu m$ thickness and mounted on gelatin-coated slides. After rapid thawing each section was fixed for 15 min in ice-cold acetone.

Ascites antibodies were diluted to $10^{-2}$ or $10^{-3}$ in PBS, and anti-*myc* was used at 2 $\mu g\,ml^{-1}$. Immunoperoxidase staining was carried out as described by Holmes *et al.* (1982), using a cascade of reagents consisting of the monoclonal antibody, rabbit anti-mouse Ig (1/1,000), swine anti-rabbit Ig (1/40), and peroxidase-rabbit antiperoxidase complex (1/80) (Dako, Copenhagen, Denmark), followed by diaminobenzidine (DAB) and hydrogen peroxide (5 mg DAB in 10 ml Tris-saline, pH 7.6, containing 0.01% $H_2O_2$). Sections were washed with Tris-saline between each reagent, and reagents were incubated for 30 min at room temperature except during the final step with DAB and $H_2O_2$, which involved a 5 min incubation at 37°C. Slides were counter-stained with Mayer's haemalum, dehydrated in alcohol, cleared in xylene and mounted in DPX (BDH Chemicals Ltd., Poole, UK). Control sections were treated with anti-human tumour monoclonal antibody 791T/36 (Embleton *et al.*, 1981) in place of the anti-oncoprotein antibodies.

### Results

*Liver lesions*

The AAF diet based on Teebor and Becker (1971) gave rise to cirrhosis, small groups of proliferating oval cells and foci of cells exhibiting raised levels of gamma-glutamyl trans-peptidase (James & Embleton, 1986). Primary liver tumours (hepatocellular carcinomas and cholangiocarcinomas) began to appear approximately one year after commencing the diet. The Solt and Farber (1976) protocol produced similar early lesions and also numerous greyish hyperplastic nodules at the liver surface within 2 months of commencing the treatment. Primary tumours developed from 7 months onwards.

*Radioimmunoassay*

Total soluble and plasma membrane extracts were tested by radioimmunoassay initially with ascites fluids diluted 1/500 and anti-*myc* antibody at 2 $\mu g\,ml^{-1}$. The results are shown in Figure 1. The highest activity was seen with anti-*ras* antibody, but it is not possible to draw firm conclusions about the relative concentrations of the different onco-proteins in a given tissue extract because the antibody content of the ascites fluid was not precisely known, or the affinities of the antibodies. The principal finding in this experiment was that there was no significant difference in binding of any of the antibodies to extracts of normal liver, liver from AAF-treated rats, or primary AAF-induced liver tumours. With most antibodies the binding to total soluble material and plasma membrane was similar but anti-*myb* bound more to total extract (which contained nuclear com-ponents) than to plasma membrane; however, bound cpm were low even with the total soluble extract. Monoclonal antibody 791T/36 gave negligible binding to either tumour or normal extracts. Similar assays were carried out with total soluble extracts of three transplanted hepatomas originally induced with 4-dimethylaminoazobenzene in comparison with two primary AAF-induced hepatomas (Figure 2). No consistent differences were seen in binding of anti-*ras*, anti-

**Figure 1** *Reactions of anti-oncoprotein monoclonal antibodies with normal liver, AAF-treated liver and primary liver tumour extracts, by radioimmunoassay.*

Total soluble extracts and plasma membrane extracts (see **Materials and methods**) were used as antigen at a concentration of 200 $\mu g\,ml^{-1}$ protein. Ascites monoclonal antibodies were diluted 1/500, and anti-*myc* was used at 2 $\mu g\,ml^{-1}$. Binding was detected using $^{125}I$-labelled anti-mouse Ig.

2AF5a, 2AF5b, 2AF6b and 2AF7 were primary AAF-induced liver tumours. 'AAF diet' refers to liver from animals taken mid-way in their fourth cycle of 0.06% AAF diet. 'Post-diet' refers to liver from rats which had completed 4 cycles of AAF diet 3 months previously. ■ plasma membrane; □ total extract.

*myc* or anti-PGDF-2 to extracts of either transplanted or primary tumours, although one primary tumour extract (2AF7) failed to bind anti-*myb*; this extract also gave no more than marginal reactivity with anti-*myb* in Figure 1.

Titrations of each antibody were performed against each of the total soluble extracts shown in Figure 1, using dilutions of ascites from $10^{-2}$ to $10^{-6}$ and anti-*myc* from 20 $\mu g\,ml^{-1}$ to 2 $ng\,ml^{-1}$. Most of the ascites preparations showed high non-specific binding to blocked plates not pretreated with antigen at $10^{-2}$ dilution, but this disappeared at $10^{-3}$. Virtually every antibody–antigen combination showed similar titration curves, a few representative examples of which are shown in Figure 3, depicting the titration of anti-*ras* and anti-*src* against normal rat liver and two primary tumour extracts. At $10^{-2}$ dilution antigen binding appeared suboptimal, perhaps due in part to a prozone effect due to antibody excess, and undoubtedly influenced by the high level of non-specific binding. From the peak at $10^{-3}$ dilution, binding titrated out at $10^{-5}$ in all cases. Anti-*myc* titrated out progressively from 20 $\mu g\,ml^{-1}$ to 20 $ng\,ml^{-1}$ (data not shown). These titrations failed to reveal any significant differences between normal liver, carcinogen-treated liver or tumour extracts at any antibody concentration.

Finally, $^{125}I$-labelled anti-*ras* was tested against tissue extracts in a direct assay. Total extract and plasma membrane produced similar results (Figure 4). Binding to normal liver and AAF-treated liver extracts was at least equal to that in specimens from four primary tumours. The specificity of the $^{125}I$-anti-*ras* antibody was confirmed by showing that binding to fixed cells of a $p21^{ras}$ expressing cell line could be inhibited by 5-fold excess unlabelled anti-*ras*, but not by 5-fold excess anti-*myc*; conversely $^{125}I$-anti-*myc* was blocked by 'cold' anti-*myc* but not by 'cold' anti-*ras*. These data are reported elsewhere (Embleton *et al.*, 1986).

### *Immunohistology*

*Liver* The staining pattern of each antibody in normal liver is indicated in Table I and illustrated in Figure 5(a)–(e). All the antibodies except anti-*src* produced peroxidase staining of hepatocytes, which although generally of no more than moderate intensity, was clearly visible. In some cases other cells within the liver (e.g. portal tracts) were also stained. Anti-*src* produced equivocal results, and was not clearly positive. Livers from AAF-treated rats, either still on the diet or three months post-treatment, showed similar staining patterns although there were structural alterations such as mild cirrhosis and isolated areas of oval cell proliferation. Hepatocytes exhibited the normal staining pattern as summarised in Table I, and cirrhotic connective tissue had the same staining characteristics as indicated for portal tracts in normal liver. Oval cells showed no clear affinity for any of the antibodies. Anti-ostegenic sarcoma antibody 791T/36 was completely negative for all tissues.

The staining of nuclei by anti-*myc* agrees with other reports (Sikora *et al.*, 1985; Watson *et al.*, 1986; Hendy-Ibbs *et al.*, 1987). However, it is surprising that this pattern was

**Figure 2** *Reactions of anti-oncoprotein monoclonal antibodies with soluble extracts of primary and transplanted rat liver tumours, by radioimmunoassay.*

Total soluble extracts were used at 200 $\mu$g ml$^{-1}$ protein concentration. Ascites antibodies were used at 1/1,000 dilution and anti-*myc* at 2 $\mu$g ml$^{-1}$. D23, D192A and D261 were transplanted rat hepatomas originally induced by oral 4-dimethylaminoazobenzene. 2AF5B and 2AF7 were primary AAF-induced tumours.

**Figure 3** *Titration of anti-oncoprotein monoclonal antibodies with soluble extracts of normal rat liver tumours, by radioimmunoassay.*

Total soluble tissue extracts were used at 200 $\mu$g ml$^{-1}$ protein concentration. Antibodies to the other oncoproteins produced similar titration profiles, although with different peak values. Other tissue extracts gave similar results to those of liver and tumours 2AF5a and 2AF6b. ●——● anti-*ras*; ■----■ anti-*src*.

**Figure 4** *Binding of* $^{125}$*I-labelled anti-*ras *monoclonal antibody to normal and AAF-treated rat liver and primary liver tumour extracts.*

Tissue extracts were used at 200 $\mu$g ml$^{-1}$ protein concentration. 'AAF diet' refers to liver from animals mid-way through a fourth cycle of 0.06% AAF diet. 2AF5a, 2AF5b, 2AF6 and 2AF7 were primary AAF-induced liver tumours. ■ Plasma membrane; □ total extract.

**Table I** Immunoperoxidase staining reactions of anti-oncoprotein monoclonal antibodies with normal rat liver

| *Antibody* | *Pattern of staining* |
|---|---|
| anti-*ras* | Moderate staining of hepatocyte plasma membrane, particularly in membrane adjacent to sinusoids. Portal tracts uniformly positive. |
| anti-PDGF-2 | Punctuate, granular staining evenly distributed in the hepatocyte cytoplasms and membrane. Portal tracts negative. |
| anti-*src* | Equivocal staining of hepatocyte plasma membrane; negative or very weak. Portal tracts negative. |
| anti-*erb*-B | Moderate staining of hepatocyte cytoplasm and portal tracts. |
| anti-*myb* | Moderate staining of hepatocyte plasma membrane and cytoplasm, but non-uniform in distribution. Nuclei stained in some cells, but not all. Portal tracts uniformly positive. |
| anti-*myc* | Hepatocyte nuclei positively stained. Moderate staining of cytoplasm adjacent to plasma membrane in some hepatocytes. Portal tracts uniformly stained. |

not consistently observed with anti-*myb*, since *myb* oncoprotein is also located in nuclei (Bishop, 1983; Heldin & Westermark, 1984). Moreover, anti-*myb* had bound to total soluble tissue extracts containing nuclear material more strongly than to plasma membrane. but anti-*myc* did not discriminate between the two types of extract (Figure 1).

*Nodules and tumours* Hyperplastic nodules and tumours mostly showed little deviation in the staining pattern of their constituent cells from that of normal liver tissue, apart from increased heterogeneity. That is, there was a tendency for variable intensity of staining within a given section, which was not apparent in normal or AAF-treated liver, but the structures stained were usually the same. There were a few exceptions however; one nodule (designated N1) showed a loss of staining by anti-PDGF-2 antibody such that only small isolated groups of cells had the punctate staining characteristic of normal hepatocytes, most of the cells being negative (Figure 5(f)). Another nodule (N2) contained cells which displayed moderate cytoplasmic staining by anti-*src* (Figure 5(g)) and cells which stained in the cytoplasm rather than the plasma membrane with anti-*ras* (Figure 5(h)). In the case of both antibodies, staining was heterogeneous and there were negative areas. One tumour with mixed elements

**Figure 5** *Immunoperoxidase staining of normal liver and carcinogen-induced hepatic nodules by anti-oncoprotein monoclonal antibodies.*

(a) Normal liver stained with anti-*myc*. Nuclei are prominent due to deposition of brown pigment, some of which is concentrated into granules within the nuclei ( x 500). (b) Normal liver stained with anti-PDGF-2. Staining of all hepatocytes has a granular, punctate appearance ( x 500). (c) Normal liver stained with anti-*src*. No dark staining visible; background staining by Mayer's haemalum only ( x 500). (d) Normal liver stained with anti-*ras*. Dark stain visible on plasma membranes adjacent to sinusoids ( x 500). (e) Normal liver stained with anti-*myb*. Generalised weak staining, with areas of darker staining on plasma membrane adjacent to sinusoids. Some nuclei stained, but others unstained ( x 500). (f) Nodule N1 stained with anti-PDGF-2. Most of the cells are negative, with isolated foci of normally stained cells; compare with (b) ( x 500). (g) Nodule N2 stained with anti-*src*. Generalised cytoplasmic staining; contrast with (c) ( x 500). (h) Nodule N2 stained with anti-*ras*. Heterogeneous staining, with areas of cells showing uniform cytoplasmic stain rather than plasma membrane localisation; compare with (d) ( x 500).

of hepatocellular carcinoma and cholangiocarcinoma (induced by the combined DENA and AAF protocol and designated DA5) showed weak uniform cytoplasmic staining with anti-*myb*, similar to that observed in normal portal tracts and in cirrhotic connective tissue in carcinogen-treated liver. these examples were the only clear differences observed in comparison with staining of normal liver.

### Discussion

Previous reports in which rats exposed to hepatocarcinogen protocols have been have been examined have indicated elevated levels of c-*ras* and c-*myc* expression in liver tumours and putatively premalignant cells. In rats fed 3′-methyl-4-dimethylaminoazobenzene, Makino *et al.* (1984) demonstrated elevated c-Ha-*ras* expression in primary tumours and also in non-tumorous parts of the liver, and elevated c-*myc* in tumours only. It was suggested that raised c-Ha-*ras* was related to hepatocyte proliferation and c-*myc* was associated with oncogenesis. In a study using three doses of DENA given after two-thirds hepatectomy, Corcos *et al.* (1984) reported raised levels of c-Ki-*ras*, c-Ha-*ras* and c-*myc* in both primary tumours and surrounding liver cells. Yaswen *et al.* (1985) have examined the expression of six oncogenes in rats fed 0.1% ethionine in a choline-deficient diet. They found that c-Ki-*ras*, c-Ha-*ras* and c-*myc* expression in the liver increased 2 weeks after starting the diet. The highest levels of c-*myc* and c-Ki-*ras* were found in oval cells, while c-Ha-*ras* was present in greater concentration in hepatocytes than in oval cells. A primary tumour was found to contain high levels of c-Ki-*ras* and c-*myc*. The c-*src* gene was expressed in the liver in detectable quantities but showed no gross changes during carcinogenesis, and c-*abl* and c-*mos* were undetectable. In all these studies oncogene expression was detected by hybridisation of extracted RNA in northern blots with labelled DNA probes.

The detection of oncoproteins by monoclonal antibody binding in our experiments did not yield comparable differences between normal liver, carcinogen-treated liver, lesions considered to be premalignant (oval cells and nodules) and primary liver tumours. Five of the six antibodies tested stained normal liver sections, and all six bound to normal liver extracts to a greater or lesser extent. Thus the oncoproteins can be considered to have been present in normal liver. Although the assays used were not strictly quantitative, increased binding or increased staining intensity should certainly have been observed within any single experiment had carcinogen-treated liver, nodules or tumours processed oncoprotein concentrations significantly above those of normal liver. That no such elevated binding was seen suggests that these tissues did not contain grossly increased amounts of oncoproteins. These findings are consistent with several reports in the literature showing that both normal and malignant cells and tissues stain with comparable intensity with anti-oncoprotein monoclonal antibodies (Kerr *et al.*, 1985; Sikora *et al.*, 1985; Williams *et al.*, 1985; Furth *et al.*, 1987; Ghosh *et al.*, 1986; Embleton *et al.*, 1986). Such observations confirm the view that oncogenes are probably very important in regulating normal cell growth and function. In some cases tumour and nodule sections showed some morphological difference in staining pattern, the principal characteristic being increased heterogeneity, and again this is commonly observed with human tumour specimens. It is doubtful if such differences are pronounced enough or consistent enough to be of diagnostic significance; certainly in our studies they could not be considered of sufficient reliability to identify the premalignant precursors of truly malignant cells.

The apparent lack of correlation between detection of oncogene-associated RNA by hybridisation and the protein products of the genes in rat liver carcinogenesis systems needs further investigation. The discrepancy may be a real one in that an RNA message is not necessarily transcribed into protein, or it may simply be a reflection of the different technologies used. One possible factor is the different carcinogen protocols, but Farber (1956) has drawn attention to the close similarities in the histological changes induced by ethionine, 3′-methyl-4-dimethylaminoazobenzene and AAF, so this seems unlikely to be cause for any major disagreement. Perhaps a fruitful area of research would be to determine tissue or cellular concentrations of oncoproteins by biochemical means not involving antibodies, and to compare these concentrations with the results of antibody binding or staining assays. Whatever the outcome of such investigations, the conclusions to be drawn from the experiments described in this paper are that currently available monoclonal antibodies to oncogene products do not consistently distinguish between normal rat liver, liver from carcinogen-treated rats, tumours or preneoplastic lesions, and that therefore they cannot be used reliably to define the precursor cells of malignant tumours.

This work was supported by the Cancer Research Campaign, London, UK. The technical assistance of Mrs A. Stibbe is acknowledged. We are grateful to Mr N.A. Habib, Dr H.L. Niman and Prof. K. Sikora for providing monoclonal antibodies.

### References

BISHOP, J.H. (1983). Cellular oncogenes and retroviruses. *Ann. Rev. Biochem.*, **52**, 301.

BROWN, J.P., WRIGHT, P.W., HART, C.E., WOODBURY, R.G. HELLSTROM, K.E. & HELLSTROM, I. (1980). Protein antigens of normal and malignant human cells identified by immunoprecipitation with monoclonal antibodies. *J. Biol. Chem.*, **255**, 4980.

CORCOS, D., DEFER, D., RAYMONDJEAN, M. & 7 others (1984). Correlated increase of the expression of the c-*ras* genes in chemically-induced hepatocarcinomas. *Biochem. Biophys. Res. Comm.*, **122**, 259.

EMBLETON, M.J., GUNN, B., BYERS, V.S. & BALDWIN, R.W. (1981). Antitumour reactions of monoclonal antibody against a human osteogenic sarcoma cell line. *Br. J. Cancer*, **43**, 582.

EMBLETON, M.J., HABIB, N.A., GARNETT, M.C. & WOOD, C. (1986). Unsuitability of monoclonal antibodies to oncogene proteins for anti-tumour drug targeting. *Int. J. Cancer*, **38**, 821.

EVAN, G.I., LEWIS, G.K., RAMSAY, G. & BISHOP, J.M. (1985). Isolation of monoclonal antibodies specific for human and mouse proto-oncogene products. *Mol. Cell. Biol.*, **5**, 3620.

FARBER, E. (1956). Similarities in the sequence of early histological changes in the liver of the rat by ethionine, 2-acetylaminofluorene and 3′methyl-4-dimethylaminoazobenzene. *Cancer Res.*, **16**, 142.

FURTH, M.E., ALDRICH, T.H. & CORDON-CARDO, C. (1987). Expression of *ras* proto-oncogene proteins in normal human tissues. *Oncogene*, **1**, 47.

GHOSH, A.K., MOORE, M. & HARRIS, M. (1986). Immunohistochemical detection of *ras* oncogene p21 product in benign and malignant mammary tissue in man. *J. Clin. Path.*, **39**, 428.

HELDIN, C.H. & WESTERMARK, B. (1984). Growth factors: Mechanism of action and relation to oncogenes. *Cell*, **37**, 9.

HENDY-IBBS, P., COX, H., EVAN, G.I. & WATSON, J.V. (1987). Flow cytometric quantitation of DNA and c-*myc* oncoprotein in archival biopsies of uterine cervix neoplastic. *Br. J. Cancer*, **55**, 275.

HOLMES, C.H., GUNN, B., AUSTIN, E.B., EMBLETON, M.J. & BALDWIN, R.W. (1982). Expression of a monoclonal antibody-defined liver-associated antigen in normal rat hepatocytes and hepatocellular carcinoma cells. *Int. J. Cancer*, **29**, 559.

HORAN-HAND, P., THOR, A., WUNDERLICH, D., MURARO, R., CARUSO, A. & SCHLOM, J. (1984). Monoclonal antibodies of predefined specificity detect activated *ras* gene expression in human mammary and colon carcinomas. *Proc. Nat. Acad. Sci.*, **81**, 5227.

JAMES, H.S. & EMBLETON, M.J. (1986). Cytotoxicity of drugs and carcinogens against hepatocytes from carcinogen-treated rats. *IRCS Med. Sci.*, **14**, 613.

KERR, I.B., LEE, F.D., QUANTILLA, M. & BALMAIN, A. (1985). Immunocytochemical demonstration of p21$^{ras}$ family oncogene product in normal mucosa and in pre-malignant and malignant tumours of the colorectum. *Br. J. Cancer*, **52**, 695.

LOWRY, O.H., ROSEBROUGH, M.L., FARR, A.L. & RANDALL, R.J. (1951). Protein measurement with the Folin phenol reagent. *J. Biol. Chem.*, **193**, 265.

MAKINO, R., HAYASHI, K., SATO, S. & SUGIMURA, R. (1984). Expressions of the c-Ha-*ras* and c-*myc* genes in rat liver tumours. *Biochem. Biophys. Res. Comm.*, **119**, 1096.

POTTER, V.R. (1978). Phenotypic diversity in experimental hepatomas; the concept of partially block ontogeny. *Br. J. Cancer*, **38**, 1.

SIKORA, K., EVAN, G.T., STEWART, J. & WATSON, J.V. (1985). Detection of the c-*myc* oncogene product in testicular cancer *Br. J. Cancer*, **52**, 171.

SOLT, D.B. & FARBER, E. (1976). New principle for the analysis of chemical carcinogenesis. *Nature*, **263**, 702.

TEEBOR, G.W. & BECKER, F.F. (1971). Regression and persistance of hyperplastic hepatic nodules induced by N-2-fluorenylacetamide and their relationship to hepatocarcinogenesis. *Cancer Res.*, **31**, 1.

THOR, A., HORAN-HAND, P., WUNDERLICH, D., CARUSO, A., MURARO, R. & SCHLOM, J. (1984). Monoclonal antibodies define differential *ras* expression in malignant and benign colonic disease. *Nature*, **311**, 562.

WATSON, J.V., STEWART, J., EVAN, G.I., RITSON, A. & SIKORA, K. (1986). The clinical significance of flow cytometric and c-*myc* oncoprotein quantitation in testicular cancer. *Br. J. Cancer*, **53**, 331.

WILLIAMS, A.R.W., PIRIS, J., SPANDIDOS, D.A. & WYLLIE, A.H. (1985). Immunohistochemical detection of the *ras* oncogene p21 product in an experimental tumour and in human colorectal neoplasms. *Br. J. Cancer*, **52**, 687.

YASWEN, P., GOYETTE, M., SHANK, P.R. & FAUSTO, N. (1985). Expression of c-Ki-*ras*, c-Ha-*ras*, and c-*myc* in specific cell types during hepatocarcinogenesis. *Mol. Cell Biol.*, **5** 780.